# The effects of simultaneous isometric and eccentric- or concentric-biased exercise on cardiovascular and muscular health of young individuals

**DOI:** 10.1007/s00421-025-06122-4

**Published:** 2026-03-07

**Authors:** A. W. Baross, B. H. Wright, D. A. Langdon, A. D. Kay, M. Cauchi, A. Clarke, B. Pitham, C. Willmont, T. Barnett, C. Key, M. Reynolds, A. Thomas, B. A. Baxter

**Affiliations:** 1 Sport and Exercise Science, University of Northampton, Northampton, NN1 5PH UK; 2 Technology, University of Northampton, Northampton, UK; 3 Health and Life Sciences, Oxford Brookes University, Oxford, UK

**Keywords:** Blood pressure, Haemodynamics, Strength, Muscle architecture, Stair ascent, Stair descent

## Abstract

**Purpose:**

Isometric resistance training (IRT) and eccentric-biased training have both demonstrated improved cardiometabolic health. Consequently, simultaneous training has the potential to further enhance these improvements and overcome the commonly reported time-related barrier to exercise. Therefore, the effects of six-week simultaneous isometric and eccentric- (stair descent) or concentric-biased (stair ascent) exercise interventions were compared to a traditional isometric handgrip training protocol on cardiovascular and muscular health parameters in 54 normotensive and hypertensive young adults.

**Methods:**

Participants (33 males, age = 21 ± 1 yr; 21 females, age = 20 ± 1 yr) were randomly assigned to four groups: simultaneous isometric exercise and stair ascent (ISO-CONC, *n* = 13), simultaneous isometric exercise and stair descent (ISO-ECC, *n* = 14), isometric handgrip (IHG) exercise (ISO, *n* = 13), or a passive control (CTRL, *n* = 14). Participants undertook thrice-weekly supervised training for six weeks, with cardiovascular and muscular function (and structure) assessed at pre- and post-training.

**Results:**

Significant reductions in resting systolic blood pressure (BP) were reported in all three training groups (*d* = 0.82–1.52), with a baseline-adjusted ANCOVA revealing that ISO-ECC diastolic BP was significantly lower than CTRL post-training. Gastrocnemius medialis muscle thickness (*d* = 0.79) and vastus lateralis fascicle angle only increased in ISO-CONC (*r* = 0.75).

**Conclusions:**

Whilst musculoskeletal improvements were only detected in the ISO-ECC group, large reductions in systolic BP were evident in all training groups, thus it may be more time-efficient to use the established isometric Handgrip intervention if reducing systolic BP is the primary therapeutic intention.

## Introduction

Cardiovascular disease (CVD) is responsible for approximately one-third of all deaths globally and is the leading cause of non-communicable disease mortality worldwide (Roth et al. 2018; WHO 2021). Hypertension (≥ 140 mmHg systolic blood pressure [SBP] and/or ≥ 90 mmHg diastolic blood pressure [DBP]; Unger et al. 2020) is a modifiable risk factor for CVD, and is reported to be near pandemic levels, with over 30% of the world’s population affected (Zhou et al. 2021). The direct costs associated with hypertension treatment in the USA is estimated to be 193 billion international dollars (Wierzejska et al. 2020) and approximately Int$2.75 billion in the UK (Fenton 2017). Although hypertension is more prevalent in older adults, the incidence of hypertension is increasing even in active younger populations (De Venecia et al. 2016; Hamrahian and Falkner 2022). Young adults tend to have less health awareness, resulting in earlier manifestation of health risks, such as diabetes or obesity (Everett and Zajacova 2015). Concernedly, those diagnosed before the age of 45 years are at greater risk (Hazard Ratio = 2.59) of CVD, hypertension, and all-cause mortality in later-life (Al Ghorani et al. 2022; Hinton et al. 2020; Suvila et al. 2019; Wang et al. 2020). Therefore, early identification of “at risk” individuals, accompanied by effective preventative or therapeutic exercise treatments is a priority as this could decrease the likelihood of developing hypertension or associated CVD in later years and reduce the global financial burden.

Isometric resistance training (IRT) has been established as one of the most efficacious non-pharmacological interventions for the prevention and treatment of hypertension (Smart et al. 2019), which elicits meaningful clinical reductions in blood pressure (BP; Wiles et al. 2017; Baross et al. 2022; Edwards et al. 2022). Moreover, eccentric-biased exercise has also demonstrated a superior ability to improve cardiometabolic health when compared to concentric-biased exercise (Chen et al. 2017a, b), despite the substantially lower metabolic and cardiovascular demand (Hoppeler 2016). Although eccentric exercise requires a low metabolic demand, it can improve muscle power, strength, and size (Douglas et al. 2017), thus it may be pivotal to developing strategies to prescribe to exercise-intolerant populations to improve cardiometabolic and muscular health throughout the lifespan. Unfortunately, eccentric-only or eccentric-biased exercises often require specialised equipment or support from a partner/trainer/clinician, hence accessibility and scalability to the wider public is often limited; however, a simple method to perform eccentric-biased exercise, is stair descent (Chen et al. 2017a, b), which has also demonstrated concomitant improvements in cardiometabolic and musculoskeletal health. Given the efficacy of isometric and eccentric training modalities to improve cardiovascular health, determining whether combining the two interventions can elicit greater improvements than already demonstrated in isolation is of practical interest and could influence clinical exercise prescription.

Training modalities can be combined either sequentially or simultaneously, although simultaneous training overcomes time-related barriers to exercise, which are commonly reported (Gee et al. 2012). Furthermore, Baross et al. (2017) reported that simultaneous (aerobic and IRT) exercise produced larger reductions in resting BP compared to single exercise protocols, hence it would appear that simultaneous exercise may be more efficacious as well as time efficient. Currently, the effects of simultaneous isometric and eccentric exercise on parameters of health remain unknown but given the ability of both to reduce BP and potentially improve muscular health simultaneously, further research is warranted in younger cohorts to delay the onset of comorbidities. Furthermore, the use of stairs to perform eccentric-biased exercise could be crucial in the development of scalable eccentric exercise prescription.

Therefore, the purpose of this study was to examine and compare the effects of a six-week simultaneous isometric and eccentric- (stair descent) or concentric-biased (stair ascent) exercise intervention to a traditional isometric handgrip intervention on cardiovascular and muscular health parameters in young adults. It was hypothesised that cardiovascular metrics would significantly change in all training groups. A secondary hypothesis was that the simultaneous training groups would also elicit improvements in muscular metrics. Furthermore, we hypothesised that objective (heart rate) and subjective (rate of perceived exertion) measures of effort during the stair walking interventions would be significantly different between groups.

## Methods

### Participants

Fifty-four normotensive (< 130/85; *n* = 44) and pre- and hypertensive (≥ 130/90; *n* = 10) adults (33 males, age = 21 ± 1 yr; 21 females, age = 20 ± 1 yr) were randomly assigned to four groups (Table 1): simultaneous isometric exercise and stair ascent (ISO-CONC, *n* = 13), simultaneous isometric exercise and stair descent (ISO-ECC, *n* = 14), isometric handgrip (IHG) exercise (ISO, *n* = 13), or a passive control (CTRL, *n* = 14). Participants did not engage in smoking or vaping and were not prescribed medication that could influence blood pressure. After receiving institutional ethical approval (Reference number, 202,201) from the University ethics review panel and prior to the first laboratory session, the participants received a detailed information sheet explaining the experimental protocol and potential risks involved, then completed and signed a physical activity readiness questionnaire (PARQ + , 2020) and informed consent form. Following a familiarisation session, which included practicing all testing, relevant intervention, and education on correct interpretation of the Category Ratio scale (CR-10, Borg 1998), pre-training data collection was completed within seven days. Participants undertook six weeks of supervised training (3 day·week^−1^), with post-intervention data collected within 24–72 h of training cessation. All data collection sessions were undertaken by the same individuals in a temperature-controlled environment (20-23ºC) at least two hours post-prandial. Participants were advised to abstain from caffeine in the 12 h prior to testing or take over-the-counter medication, undertake vigorous exercise or consume alcohol for 24 h preceding the data collection sessions.

**Table 1:** Participant pre-training demographic and blood pressure data

Group	CTRL	ISO	ISO-CONC	ISO-ECC
Age (yr)	21 ± 1	21 ± 1	21 ± 1	20 ± 1
Height (cm)	175 ± 6	167 ± 8	168 ± 11	175 ± 7
Body Mass (kg)	75 ± 14	74 ± 18	72 ± 20	78 ± 15
*Resting Blood Pressure*				
SBP (mmHg)	121 ± 13	118 ± 10	118 ± 9	123 ± 13
DBP (mmHg)	65 ± 8	73 ± 6	62 ± 7	67 ± 10
MAP (mmHg)	84 ± 9	88 ± 7	81 ± 6	85 ± 10

Values are mean ± SD. *ISO* isometric group, *ISO-ECC* simultaneous isometric and eccentric training group, *ISO-CONC* simultaneous isometric and concentric training group, *CTRL* control group, *SBP* systolic blood pressure, *DBP* diastolic blood pressure, *MAP* mean arterial pressure.

## Procedures

### Cardiovascular metrics

#### Haemodynamics

Continuous and non-invasive blood pressure monitoring (CNAP^®^500, CNSystems Medizintechnik AG, Austria) was used to measure resting cardiac output (Q; L·min^−1^), stroke volume (SV; mL) and systemic vascular resistance (SVR; dyn·s^−1^·cm^−5^). The CNAP® monitor used a double-adjacent finger cuff placed on the left hand, with a standard oscillometric blood pressure cuff on the upper right arm to allow for calibration to brachial pressure. Participants were seated in a chair, with legs uncrossed and knees at 90° flexion (180° = knee extension), with the left arm supported at approximately heart hight and the right arm supported on the participant’s thigh. The double finger cuff was placed around the 3rd and 4th proximal joints of the metacarpal of the index and middle phalange with the attached CNAP® controller placed on the participant’s forearm by Velcro fixation. Participants were asked to sit still and in silence for a 10-min period, with resting beat-to-beat measures continuously recorded during the final 5-min. Immediately after the end of the 10-min period, resting SBP (mmHg), DBP (mmHg), mean arterial pressure (MAP; mmHg) and heart rate (HR; beat·min^−1^) were measured using an automatic blood pressure monitor (UA-767 Plus, A&D Company, Ltd., Tokyo, Japan). The blood pressure cuff was placed contralaterally on the participant’s upper left arm (above brachial pulse), with three measurements taken at 1-min intervals and the mean from the three trials used for subsequent analysis.

#### Cardiometabolic markers

Fasted capillary blood samples (35 *μ*L) were collected from the index or middle finger with a single-use lancet (Accu-Chek® safe T plus, Roche Diabetes, Portal) for the measurement of fasting blood glucose (TEE2 + , i-SENS, Inc. Seoul, Korea) and full lipid profiles, including total cholesterol, high density lipoproteins (HDL), low density lipoproteins (LDL) and triglycerides (Mission^®^ ACON Labs Inc, USA). All samples were taken in accordance with established guidelines and collected at a constant room temperature between 20 and 23 °C.

### Muscular metrics

#### Muscle structure

In vivo muscle structure of the *vastus lateralis* (VL) and the medial head of the *gastrocnemius medialis* (GM) was examined using a real-time 2-dimensional B-mode ultrasonography (Vivid I, General Electric, Bedford, UK) with a wide band linear probe (8 L-RS, General Electric) using a scanning frequency of 10 MHz and coupling gel (Ultrasound gel, Dahlhausen, Cologne, Germany) between the probe and skin.

For imaging of the VL, participants lay supine on a therapy bed with the probe positioned over the mid-point of the thigh between the lateral femoral condyle and greater trochanter. For imaging of the GM, participants lay in a prone position with feet hanging over the therapy bed, with the probe positioned one third proximal between the popliteal crease and calcaneus. The probe was manipulated over the VL and GM to visualise the superficial and deep aponeuroses and rotated until the muscle fascicles also became clear. An image was then captured before the probe was removed and then replaced in the same area to capture a second image so that the mean of the two images could be calculated and subsequently analysed.

### Automated measurements

An interactive graphical user interface (GUI; DL_Track_US [v0.2.1]) developed in the Python language [v3.10] (Ritsche et al. 2023) was downloaded and installed on a HP 45L Gaming Desktop PC utilising an Intel-i9 13th Gen CPU, 64 GB RAM, and Nvidia GeForce RTX 4090 with 24 GB GPU RAM. The automated analysis involves the importation of two convolution neural network models both employing a VGG16 encoder path, which focus on muscle fascicles and aponeuroses. The GUI can import several different image formats such as jpg, tiff, png, and bmp; in this study, all images were imported as jpg. The default parameters recommended by the authors of the GUI were used. An Excel file was used to convert the pixel values into millimetres (mm) prior to statistical analysis; where it was observed that there were blank cells in the Excel file, for example only the Muscle Thickness (“Midthick”) value was attained by the GUI, the corresponding image file was processed manually (described below). The validity of this method, specifically including analysis of the GM and VL architecture has been previously reported (Cronin et al. 2020) with mean differences between the automated and manual method of 2.1 mm, 0.1°, and 0.1 mm reported for fascicle length, fascicle angle, and muscle thickness, respectively.

### Manual measurements

Sonographs were exported to a manual digitisation software for further analysis (ImageJ 1.46r, National Institutes of Health, Bethesda, MD, USA). For muscle thickness, six measurements of the VL and GM were measured as the distance between the superficial and deep aponeuroses from both images, with the mean used for subsequent analysis (Baxter et al. 2024). Fascicle angle was determined using the insertion angle of the muscle fascicles into the deep aponeurosis, where three measures were taken, and the mean was used for analysis. As fascicles were too long to measure directly from the sonograph, standard trigonometry was used to estimate fascicle length to align with the calculations of the automated method detailed above.

#### Strength

Maximum IHG strength (lb) was assessed using a digital handheld dynamometer (ZONA Plus 3, FL, USA) by performing three maximum voluntary contractions (MVC) for each hand, with a 30-s rest between contractions and assessed following the American Society of Hand Therapists standardised testing position (Roberts et al. 2011). Whilst in the standing position, participants were instructed to hold the dynamometer with the elbow flexed to 90° (180° = full elbow extension), while keeping the elbow close to the body with the forearm in a neutral position. The participants were instructed to grip the dynamometer as “*hard”* as they could (~ 5 s) before switching hands. A practice trial was provided on each hand before a further three measurement trials were taken. The greatest value of the three trials was taken and used for subsequent analysis.

Isokinetic dynamometry was used to assess maximum voluntary isometric knee extensor torque (N·m). The participants sat on the dynamometer chair (Biodex system 3 Pro, medical systems Inc. NY, USA) with the hips flexed at 95° (180° = full extension) and right knee flexed at 110°. The right lateral femoral condyle was aligned to the axis of rotation of the dynamometer with the right shank placed into the leg attachment and securely tightened just proximal to the ankle. Participants performed three sub-maximal isometric contractions at their perceived 50% maximum, separated by 15 s of rest between repetitions with arms folded and the thigh strapped to the dynamometer; the same process was conducted for 75% of the participants’ perceived effort. Subsequently, participants were asked to perform three maximal ramped contractions by building up to a maximal effort over a 5-s window, with verbal instruction provided by the researcher throughout. If any countermovement was evident (visual check for an initial reduction in torque), then the contraction data were not used and the test was repeated. The greatest torque of the five trials was used for subsequent analysis.

### Training protocol

#### Simultaneous isometric handgrip and stair ascent/descent interventions

During the first week of training, participants completed nine repetitions of either stair ascent or descent in all three sessions, with one repetition consisting of 108 steps (vertical displacement = 17.8 m). The training volume increased weekly by increasing the number of stair repetitions (9, 12, 15, 18, 21, 24). To avoid eccentric work in the ISO-CONC group and concentric work in the ISO-ECC group, an elevator was used to return the participant to the top or bottom of the stairs. The walking tempo was set at approximately 1 step·s^−1^, taking ~ 2 min to complete one repetition of stair ascending or descending, including steps on each landing and the short walk to the elevator; a further 2 min (elevator time) elapsed before the next stair repetition commenced. Both groups completed four simultaneous, 2-min handgrip contractions, evenly interspaced throughout the training session using alternate hands. IHG exercise was undertaken using a commercially available adjustable IHG device with variable resistance. The participants were asked to squeeze the IHG device at an intensity equivalent to a perceived rate of exertion (CR-10 scale) of “4” for the first min, rising to “5” for the second min (Wright et al. 2025). To ensure participants understood the “*maximal*” sensation on the CR-10 scale and provide a reference point for the handgrip exercise intensity, an exercise anchoring procedure (Lagally and Costigan 2004) was completed during familiarisation; to achieve this, participants were required to complete a 3-s maximal handgrip contraction.

#### Isometric handgrip intervention

The ISO group undertook the same IHG protocol with 2 min recovery between each handgrip contraction that replicated as close as possible to the 4 × 2-min contractions undertaken simultaneously with the stair training, again starting at a perceived rate of exertion (CR-10 scale) of “4” rising to “5” for the second min of the contraction. At the end of each training session, participants’ rate of perceived exertion (RPE), muscle soreness, and sessional HR were recorded. CTRL attended the laboratory on three occasions (familiarisation, pre-training, and post-training) and were asked to maintain their normal physical activity levels over the 6-week period.

#### Sessional heart rate and rate of perceived exertion

Sessional HR was recorded using chest-strapped monitors (Polar H10, Polar Electro, Kempele, Finland) and recorded as the mean HR throughout the training session, with recording started prior to the first repetition until the end of the final repetition. The mean of the three weekly training sessions was calculated and used for subsequent analysis. Similarly, RPE was recorded at the end of each training session using a 15-point scale (6–20; Borg 1998) with the mean of the three weekly sessions used for subsequent analysis.

### Statistical analysis

Statistical analyses were conducted using SPSS for Windows (Version 28, IBM Corp., Armonk, NY, USA). All data were initially screened for normality (Shapiro-Wilks test) and homogeneity of variance (Levene’s or Mauchly Tests). Data that failed assumptions of normal distribution were transformed; if data continued to fail to meet the assumption of normal distribution, then non-parametric tests were used to test for between- (Kruskal–Wallis test) or within-group (Wilcoxon tests) differences. One-way ANOVAs (parametric) or Kruskal–Wallis tests (non-parametric) were conducted to assess any significant between-group differences that occurred at baseline to determine whether baseline data needed to be considered as a covariate. Data that satisfied the parametric assumptions were assessed via a series of factorial ANOVAs for time × group to examine interaction, within-, and between-subject effects; ISO was not included in the statistical analysis of muscular structure given that the lower-limb musculature was not targeted in the training programme. If sphericity was violated where epsilon (ε) was > 0.75 then a Huynh–Feldt correction was used and if ε was < 0.75 then a Greenhouse–Geisser correction factor was used (Field 2009, p.850). If a significant interaction effect was detected, then post-hoc simple main effects analysis (pairwise comparisons) was conducted with a Bonferroni correction. Where no significant interaction was detected the data sets were collapsed with the main effects of time and group analysed. A factorial ANOVA with six levels of time (week 1…6) was conducted for sessional HR, with Tukey’s honestly different test used to assess post-hoc pairwise comparisons. For non-parametric analyses, the standardised magnitude of change, *r* was calculated, with partial eta squared (ƞ_p_^2^) and Cohen’s *d* calculated for parametric analyses. All group mean and variation data are presented as mean and standard deviation (SD) for data that satisfied parametric assumptions or median and interquartile range (IQR) for data that did not satisfy parametric assumptions.

## Results

### Training metrics

#### Heart rate

**Fig. 1:**
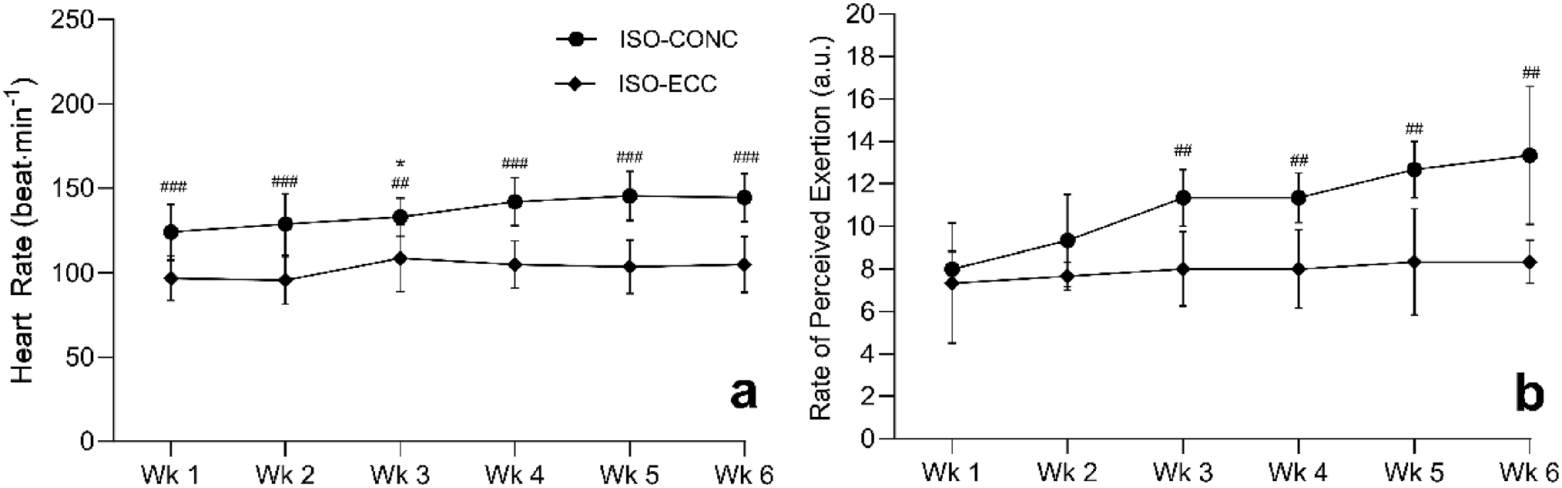
Mean ± SD exercising heart rate (**a**) and rate of perceived exertion (**b**) during six weeks of simultaneous isometric resistance training and concentric- (ISO-CONC) or eccentric-biased (ISO-ECC) exercise. ## denotes a between-group difference *P* < 0.01, ### *P* < 0.001, and * denotes a significant within-group difference compared to the previous week within ISO-ECC *P* < 0.05. Heart rate was significantly greater (24–42 beat·min^−1^) in ISO-CONC compared to ISO-ECC during all six weeks of training, with a consecutive weekly significant increase (13 beat·min^−1^) in heart rate only evident within ISO-ECC from week 2 to week 3. Rate of perceived exertion was significantly greater in ISO-CONC than ISO-ECC from week 3 to week 6 (2–4 a.u.)

A significant interaction effect was revealed for HR during training (*F*_2.983, 56.686_ = 395.620, *P* = 0.042, η_p_^2^ = 0.133), with HR significantly greater in ISO-CONC than ICO-ECC during each week of training (*P* < 0.05, *d* = 1.50–2.73). When comparing HR at consecutive weeks, a significant increase was only evident in ISO-ECC from week 2 to week 3 (*P* = 0.004), see Fig. 1a.

#### Rate of perceived exertion

Mann–Whitney U tests revealed that RPE was significantly greater (*P* < 0.01) in ISO-CONC than ISO-ECC from week 3 to week 6. Wilcoxon tests revealed no significant increase in RPE within either group when comparing consecutive weeks (*P* > 0.05), see Fig. 1b.

### Cardiovascular metrics

#### Resting blood pressure

**Fig. 2:**
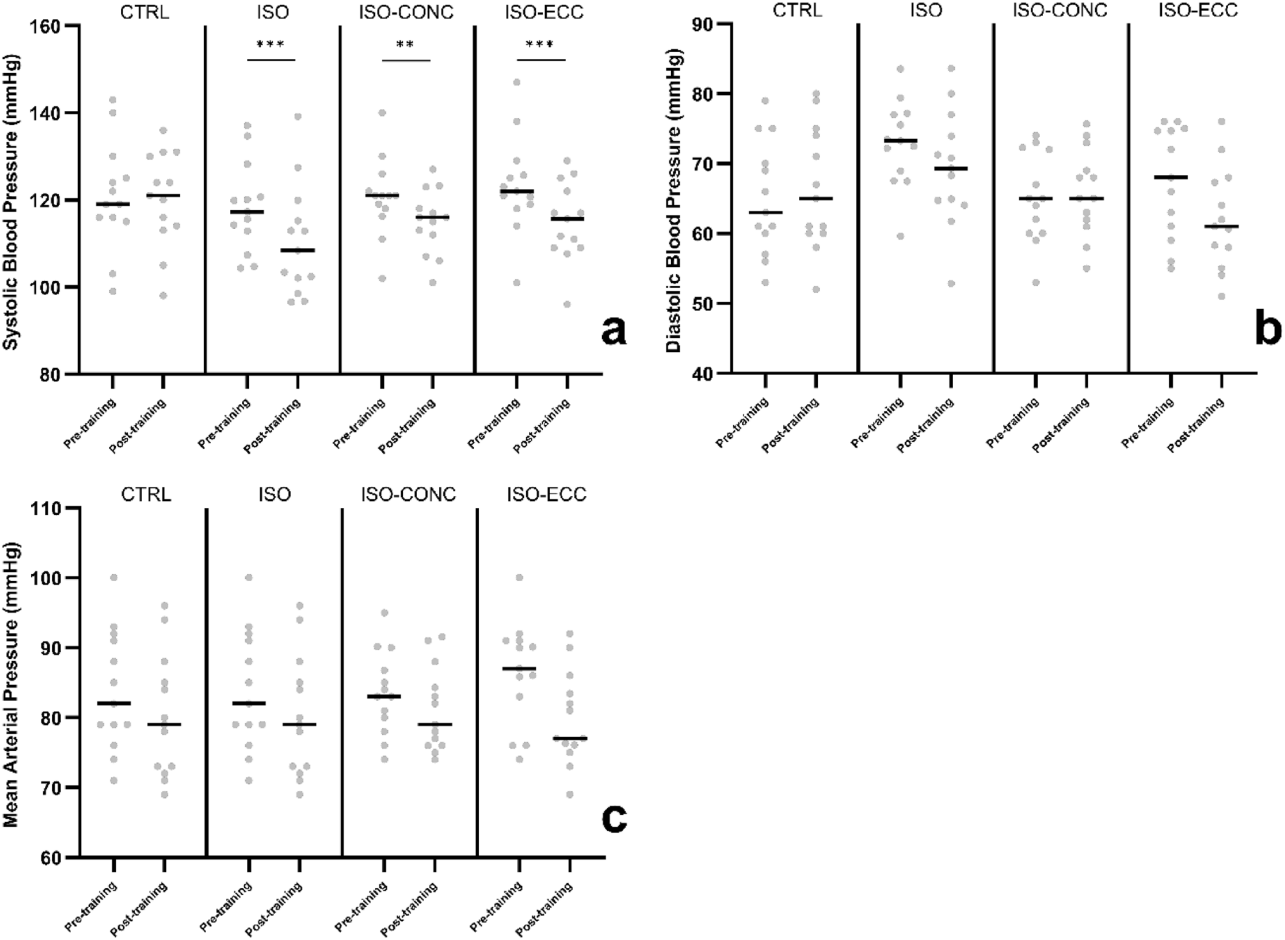
Mean (lines) and individual (dots) systolic blood pressure (**a**), diastolic blood pressure (**b**)—raw unadjusted diastolic blood pressure values are displayed, hence symbols that denote significant differences have not been included, and mean arterial pressure (**c**) before and after the six weeks of simultaneous isometric resistance training and concentric- (ISO-CONC) or eccentric-biased (ISO-ECC) exercise, isometric handgrip training (ISO), or no training (CTRL). ** denotes a within-group difference *P* < 0.01 and *** *P* < 0.001. Significant reductions in systolic blood pressure were evident within ISO (8 ± 9 mmHg), ISO-CONC (6 ± 4 mmHg), and ISO-ECC (8 ± 5 mmHg), whereas no significant difference was revealed within CTRL (1 ± 8 mmHg)

A significant interaction effect was revealed for SBP (*F*_3, 48_ = 3.198, *P* = 0.032, η_p_^2^ = 0.167), with significant reductions (*P* < 0.05) revealed in all training groups (∆ ISO = 8 ± 9 mmHg,* d* = 0.82; ISO-CONC = 6 ± 4 mmHg*, d* = 1.23; ISO-ECC = 8 ± 5 mmHg, *d* = 1.52) but not CTRL (1 ± 8 mmHg, *d* = 0.08); no significant between-group differences were detected at either time point (*P* > 0.05), see Fig. 2a. Regarding DBP, a one-way ANOVA revealed a significant effect of group at pre-training (*F*_3, 48_ = 3.454, *P* = 0.024, η_p_^2^ = 0.178), with ISO significantly greater than CTRL (*P* = 0.044, *d* = 1.10) and ISO-CONC (*P* = 0.048, *d* = 1.25), therefore an ANCOVA was conducted with pre-training as the covariate. The ANCOVA revealed a significant group effect post-training (*F*_3, 47_ = 3.682, *P* = 0.018, η_p_^2^ = 0.190), with ANCOVA adjusted DBP in ISO-ECC (62 ± 5 mmHg) significantly lower than CTRL (68 ± 5 mmHg),

No significant interaction effect was revealed for MAP (*F*_3, 48_ = 1.150, *P* = 0.339, η_p_^2^ = 0.067), with a main effect of time (*F*_1, 48_ = 28.398, *P* < 0.001, η_p_^2^ = 0.372) but not group (*F*_3, 48_ = 0.892, *P* = 0.452, η_p_^2^ = 0.053) detected. Data collapsed across groups revealed a significant decrease (mean ± SD ∆ = 4 ± 6 mmHg; *P* < 0.001, *d* = 0.74) in MAP over time, see Fig. 2c.

#### Cardiac output, stroke volume, and systemic vascular resistance

No significant interaction effects were revealed for cardiac output, stroke volume, or systemic vascular resistance (*F*_3, 48_ = 0.635–1.606, *P* = 0.201–0.596, η_p_^2^ = 0.039–0.093), alongside no main effects of time (*F*_1, 48_ = 0.325–1.659, *P* = 0.204–0.571, η_p_^2^ = 0.007–0.034) nor group (*F*_3, 48_ = 0.170–0.550, *P* = 0.651–0.916, η_p_^2^ = 0.011–0.034). Wilcoxon and Mann–Whitney U tests revealed no significant (*P* > 0.05) within- or between-group differences for HR, see Table 2.

**Table 2:** Haemodynamic and cardiometabolic metrics at pre- and post-training

**Group**	CTRL	ISO	ISO-CONC	ISO-ECC
*Variable*				
*HR* (beat·min^−1^) Pre-training Post-training	72 (23) 73 (15)	75 (16) 76 (10)	75 (5) 75 (10)	75 (18) 73 (15)
*SV* (mL) Pre-training Post-training	96.9 ± 14.4 92.5 ± 18.2	92.0 ± 15.5 86.9 ± 14.4	88.9 ± 14.2 88.8 ± 19.2	93.0 ± 17.6 94.7 ± 15.1
*Q* (L·min^−1^) Pre-training Post-training	7.1 ± 1.5 6.8 ± 1.2	6.7 ± 1.1 6.9 ± 1.3	7.1 ± 0.8 6.9 ± 1.1	6.9 ± 1.0 6.7 ± 1.2
*SVR* (dyn·s^−1^·cm^−5^) Pre-training Post-training	966 ± 244 1034 ± 252	981 ± 149 953 ± 180	925 ± 131 998 ± 185	1031 ± 253 978 ± 255
*Cholesterol* (mg·dL^−1^) Pre-training Post-training	166 ± 38 180 ± 28	177 ± 30 171 ± 39	171 ± 55 172 ± 44	158 ± 31 152 ± 29
*HDL* (mmol·L^−1^) Pre-training Post-training	39.6 ± 12.6 47.0 ± 21.1	58.9 ± 10.3 55.8 ± 15.4	43.1 ± 16.0 49.6 ± 20.1	43.9 ± 14.8 44.5 ± 13.0
*LDL* (mmol·L^−1^) Pre-training Post-training	101.4 ± 17.8 105.3 ± 19.3	96.2 ± 27.5 103.0 ± 43.6	89.7 ± 35.7 93.1 ± 37.5	93.7 ± 26.3 88.3 ± 21.5
*LDL/HDL ratio* Pre-training Post-training	3.85 (1.77) 4.50 (2.97)	2.95 (1.25) 2.85 (1.60)	3.70 (1.60) 3.20 (1.43)	3.70 (1.50) 3.50 (1.40)
*Triglycerides* (mg·dL^−1^) Pre-training Post-training	97.5 (58.2) 121.5 (116.8)	107.5 (72.0) 65.5 (49.5)	114.5 (90.8) 106.0 (85.5)	92.0 (63.0) 91.0 (39.0)
*Glucose* (mmol) Pre-training Post-training	5.35 (0.57) 5.55 (1.23)	5.75 (0.68) 5.25 (0.43)	5.75 (1.45) 5.35 (0.35)	5.10 (0.50) 5.30 (1.00)

Metrics that satisfied the parametric assumptions are displayed as means ± SD, whereas values that did not satisfy parametric assumptions are displayed as median (IQR); *CTRL* control group, *ISO* isometric group, *ISO-CONC* simultaneous isometric and concentric-biased group, *ISO-ECC* simultaneous isometric and eccentric-biased group, *HR* resting heart rate, *SV* stroke volume, *SVR* systemic vascular resistance, *HDL* high density lipoprotein, *LDL* low density lipoprotein.

#### Cardiometabolic markers

No significant interaction effects were revealed for cholesterol, HDL, or LDL (*F*_3, 44_ = 0.448–2.022, *P* = 0.125–0.720, η_p_^2^ = 0.030–0.121), alongside no main effects of time (*F*_1, 44_ = 0.002–1.941, *P* = 0.171–0.962, η_p_^2^ = 0.000–0.042) nor group (*F*_3, 44_ = 0.662–2.242, *P* = 0.097–0.580, η_p_^2^ = 0.043–0.133). Wilcoxon and Mann–Whitney U tests revealed no significant (*P* > 0.05) within- and between-group differences for Triglycerides, LDL/HDL ratio, or blood glucose, see Table 2.

### Muscular outcome measures

#### Strength

A one-way ANOVA revealed a significant effect of group at pre-training for dominant and non-dominant handgrip strength (*F*_3, 42_ = 4.365–5.023, *P* = 0.005–0.009, η_p_^2^ = 0.238–0.264), with ISO dominant handgrip strength significantly lower than CTRL (*P* = 0.041, *d* = 1.35) and ISO-ECC (*P* = 0.014, *d* = 1.32), see Table 3. Non-dominant handgrip strength within ISO was significantly lower than CTRL (*P* = 0.020, *d* = 1.41), ISO-CONC (*P* = 0.046, *d* = 1.22), and ISO-ECC (*P* = 0.011, *d* = 1.33). Therefore, ANCOVA’s were conducted with pre-training handgrip strength as the covariate. The ANCOVA revealed a significant effect of group at post-training for dominant handgrip strength (*F*_3, 41_ = 2.934, *P* = 0.045, η_p_^2^ = 0.177), with ANCOVA adjusted handgrip strength significantly (*P* = 0.049, *d* = 0.57) greater in ISO (86.0 ± 7.9 lb) than ISO-CONC (77.2 ± 7.3 lb). For non-dominant handgrip strength, the ANCOVA revealed no significant effect of group at post-training (*F*_3, 41_ = 2.264, *P* = 0.095, η_p_^2^ = 0.142). No significant interaction effect was revealed for maximal isometric knee extensor torque (*F*_2, 37_ = 0.113, *P* = 0.894, η_p_^2^ = 0.006), alongside no main effects of time (*F*_1, 37_ = 3.978, *P* = 0.054, η_p_^2^ = 0.097) or group (*F*_2, 37_ = 0.850, *P* = 0.436, η_p_^2^ = 0.044).

**Table 3:** Muscular strength and structure metrics at pre- and post-training

Group	CTRL	ISO	ISO-CONC	ISO-ECC
*Variable*				
*Knee extensor torque (N·m)* Pre-training Post-training	72 (64, 87) 73 (60, 75)	75 (69, 53) 76 (74, 64)	75 (71, 76) 75 (69, 79)	75 (62, 80) 73 (67, 82)
*D IHG strength (pounds)* Pre-training Post-training	84.4 ± 19.4 # 84.9 ± 18.2	62.4 ± 13.7 73.5 ± 9.2	80.1 ± 18.2 79.2 ± 18.7 #	86.3 ± 22.0 # 85.5 ± 19.8
*ND IHG strength (pounds)* Pre-training Post-training	81.5 ± 20.1 # 78.4 ± 22.9	57.3 ± 13.3 67.1 ± 9.8	78.1 ± 18.8 # 78.0 ± 19.1	82.6 ± 22.7 # 83.5 ± 22.1
*VL muscle thickness (mm)* Pre-training Post-training	23.5 ± 4.3 22.6 ± 4.1	NA NA	21.4 ± 3.1 21.4 ± 2.5	22.3 ± 4.6 22.9 ± 4.4
*VL fascicle angle (°)* Pre-training Post-training	14.9 (7.5) 13.6 (6.1)	NA	15.2 (5.1) 15.4 (8.5) *	16.4 (4.4) 15.0 (4.8)
*VL fascicle length (mm)* Pre-training Post-training	88.5 ± 30.4 77.8 ± 26.3	NA	83.9 ± 20.0 70.2 ± 17.2	88.7 ± 32.5 101.1 ± 51.7
*GM muscle thickness (mm)* Pre-training Post-training	19.8 ± 2.9 18.9 ± 3.2	NA	16.5 ± 3.6 19.0 ± 3.2 **	16.8 ± 3.4 17.8 ± 2.8
*GM fascicle angle (*°*)* Pre-training Post-training	24.7 ± 4.9 26.5 ± 2.8	NA	23.0 ± 7.5 24.0 ± 4.6	21.0 ± 2.9 21.6 ± 3.3
*GM fascicle length (mm)* Pre-training Post-training	50.0 ± 12.2 39.8 ± 8.4	NA	46.4 ± 18.0 46.4 ± 10.8	48.7 ± 12.4 48.3 ± 6.4

Metrics that satisfied the parametric assumptions are displayed as means ± SD, whereas values that did not satisfy parametric assumptions are displayed as median (IQR); *CTRL* control group, *ISO* isometric group, *ISO-CONC* simultaneous isometric and concentric-biased group, *ISO-ECC* simultaneous isometric and eccentric-biased group. *D IHG* dominant isometric handgrip strength; *ND IHG* non-dominant isometric handgrip; *VL* vastus lateralis; *GM* gastrocnemius medialis, # denotes a between-group difference to ISO at the respective time point to *P* < 0.05, * denotes a significant within-group difference between time points to *P* < 0.05 and ** to *P* < 0.01; note – post-training handgrip values are ANCOVA adjusted means.

#### Muscle structure

A significant interaction effect was revealed for GM muscle thickness (*F*_2, 34_ = 3.873, *P* = 0.031, η_p_^2^ = 0.186), with a significant increase only observed in ISO-CONC (18.2 ± 25.0%, 0.26 ± 0.29 mm; *P* = 0.007, *d* = 0.79); no significant between-group differences were observed at either time point, see Table 3. No significant interaction effect was revealed for VL muscle thickness (*F*_2, 34_ = 1.040, *P* = 0.364, η_p_^2^ = 0.058), alongside no main effects of time (*F*_1, 34_ = 0.069, *P* = 0.794, η_p_^2^ = 0.002) or group (*F*_2, 34_ = 0.622, *P* = 0.543, η_p_^2^ = 0.035).

Wilcoxon tests revealed a significant increase in VL fascicle angle post training in ISO-CONC (3.5 ± 4.5°, *P* = 0.013, *r* = 0.75), with no significant changes in any other group (*P* > 0.05); Kruskal–Wallis tests revealed no significant between-group differences at pre-training (c2^[37]^ = 0.041, *P* = 0.980) or post-training (c2^[37]^ = 4.164, *P* = 0.125). No significant interaction effect was revealed for GM fascicle angle (*F*_2, 34_ = 0.269, *P* = 0.766, η_p_^2^ = 0.016), alongside no main effect of time (*F*_1, 34_ = 2.908, *P* = 0.097, η_p_^2^ = 0.079), however a significant main effect of group was detected (*F*_2, 34_ = 3.680, *P* = 0.036, η_p_^2^ = 0.178), with GM fascicle angle significantly greater in ISO-ECC than CTRL (*P* = 0.031, *d* = 1.11) at post training.

No significant interaction effect was revealed for VL or GM fascicle length (*F*_2, 34_ = 2.688–3.022, *P* = 0.062–0.084, η_p_^2^ = 0.136–0.151), alongside no main effects of time (*F*_1, 34_ = 0.000–1.566, *P* = 0.219–0.999, η_p_^2^ = 0.000–0.044) or group (*F*_2, 34_ = 0.732–1.358, *P* = 0.271–0.488, η_p_^2^ = 0.041–0.074).

## Discussion

This is the first study to investigate the effects of simultaneous isometric and eccentric- or concentric-biased exercise on cardiovascular and muscular health of young adults. All three training modalities were efficacious therapeutic exercise interventions that lowered SBP, with ISO-ECC also reducing DBP. If SBP reduction is the main goal of the therapeutic treatment, it may be more appropriate and time efficient to use the established isometric handgrip intervention (ISO). Although ISO-CONC resulted in improvements in lower-limb muscle structure, albeit without improvements in strength, it was objectively and subjectively harder to perform compared to ISO-ECC.

To counter the considerable number of adults not meeting physical activity guidelines (Millar et al. 2007; Sandercock et al. 2022), exercise recommendations suggest interventions be accessible, inexpensive, and inclusive for those with reduced functional ability (Heath et al. 2012; Tuso 2015; Collado-Mateo et al. 2023). Given the ability to incorporate stair walking into daily living, it is an inexpensive and accessible form of exercise (Takaishi et al. 2014) that elicits significant cardiovascular health benefits (Ghosal and Chandrasekaran 2024), including improved cardiorespiratory fitness (Allison et al. 2017), metabolic profile (Whittaker et al. 2021), and blood pressure (Andersen et al. 2013). In the present study, both stair walking training groups demonstrated significant reductions in resting SBP (Fig. 2), findings consistent to those of Chen et al. (2017a, b) but in a young population where the prevalence of hypertension is increasing (Meher et al. 2023). Unlike Chen et al. (2017a, b) who reported significantly greater SBP reductions following stair descent (eccentric-biased; ~ 11 mmHg) than ascending (concentric-biased; ~ 4 mmHg), no stair training group was superior in reducing resting SBP. Given that cardiovascular and perceptual demand were lower in ISO-ECC than ISO-CONC, stair descent may be a preferable intervention to prescribe to exercise-intolerant cohorts.

As all training groups elicited large clinically meaningful (Kandzari et al. 2022) reductions in SBP (*d* = 0.82–1.52), the prescription of IHG in isolation may be a more suitable exercise intervention in time- and mobility-limited populations as it does not necessitate lower-limb function. Furthermore, as a portable and inexpensive (~ £8) device was used to induce the adaptations, the ISO programme also overcomes logistical and financial barriers, which increases the accessibility and scalability of the intervention. The SBP reductions following ISO are consistent with previous IHG studies (Millar et al. 2008; Garg et al. 2014; Badrov et al. 2016), even when using a commercially available device (Millar et al. 2008), further substantiating the antihypertensive effects of IRT (Edwards et al. 2024). The present study also reinforces the use of RPE to adequately control isometric contraction intensity (Lea et al. 2021; Wright et al. 2025). An important observation of the present study is that compared to studies of stair walking performed in isolation (Meyer et al. 2010; Andersen et al. 2013) that reported reductions in SBP (1–3 mmHg), the implementation of IHG adjunctively with stair walking resulted in larger absolute decreases in resting SBP (6–8 mmHg). Comparable disparate changes were also detected by Baross et al. (2017), who reported simultaneous IHG and walking (no incline or decline) produced significantly larger SBP reductions (10 mmHg) than IHG (5 mmHg) or walking (5 mmHg) when undertaken individually. Notably, these SBP reductions are comparable to those observed after eight weeks of isometric (Inder et al. 2016) and aerobic (Cornelissen and Smart 2013) exercise, though achieved after only six weeks. Thus, as stair climbing interventions demonstrate improvements in cardiometabolic health (Ghosal and Chandrasekaran 2024) and lower extremity function (Kim et al. 2024), incorporating simultaneous IHG may represent a more favourable and time-efficient therapeutic approach than exercise modalities performed in isolation.

The assessment of haemodynamic parameters in the present study provides a mechanistic insight into potential pathways for BP reduction following exercise intervention (Li et al. 2023). Stair walking interventions have been shown to elicit positive adaptations to cardiovascular function and structure (Cho et al. 2020; Yamaji et al. 2021), accompanied by improvements in inflammatory profile and body composition (Chow et al. 2021). However, in the present study BP reductions occurred across each training group without significant changes to haemodynamic measures or cardiometabolic risk markers, which is consistent with the findings of Kirk et al. (2025). These disparate findings may be explained by participants in the present study being young adults with a low cardiovascular risk profile and limited capacity for cardiovascular adaptation, to the extent that the magnitude of reduction may be inhibited by regulatory mechanisms preventing values to fall below homeostatic ranges. Additionally, as haemodynamic measures were examined using a non-invasive analogue device, although reliable and valid for beat-to-beat arterial blood pressure measurement (Jeleazcov et al. 2010; Jagadeesh et al. 2012), the accuracy and validity of non-invasive pulse contour analysis for the assessment of haemodynamic measures are disputed (Roth et al. 2018).

Whilst stair walking studies have evidenced a reduction in DBP (Meyer et al. 2010; Andersen et al. 2013), in the present study this reduction only occurred in the ISO-ECC group, which is somewhat surprising given the consistently lower cardiovascular demand when compared to ISO-CONC, although this has also been reported following the prescription of a home-based eccentric-biased programme (Kirk et al. 2025). It is possible that eccentric-biased exercise augments nitric oxide production and bioavailability (Sakellariou et al. 2021) in response to impaired microvascular reactivity (Larsen et al. 2019) and the formation of reactive oxygen species during eccentric exercise (He et al. 2016; Schieber and Chandel 2014). Moreover, alterations in flow-mediated dilation (Cho et al. 2020; Yamaji et al. 2021) have been reported following stair walking interventions and in an acute setting, eccentric contractions have demonstrated greater autonomic function responses compared to concentric or isometric (Gois et al. 2014). Thus, completing simultaneous eccentric and isometric exercise may elicit a unique cardiovascular stimulus sufficient to reduce DBP in normotensive adults, which is important given isolated diastolic hypertension is a risk factor among younger adults (McEvoy et al. 2021). However, further investigation is required to confirm these findings, the potential underlying mechanisms, and whether these results may be influenced by participant clinical demographics, and inter-individual variations in response to exercise.

Regarding muscular adaptations, significant changes in muscle structure only occurred in ISO-CONC, with significant increases evident in VL fascicle angle and GM muscle thickness. Increases in fascicle angle and mid-belly muscle thickness are commonly observed following concentric exercise (Franchi et al. 2017), which contribute towards an increase in physiological cross-sectional area, a key determinant of force production (Lieber and Friden 2000). However, despite the increase in VL fascicle angle, no significant change in VL thickness or knee extensor strength were evident. The lack of structural changes within ISO-ECC are somewhat unexpected (Franchi et al. 2017) but may be explained by methodological limitations. For example, eccentric-specific exercise tends to elicit increases in distal muscle thickness as opposed to mid-belly thickness (Franchi et al. 2017). Furthermore, trigonometric functions do not account for site-specific adaptation or the curvature of fascicles but show strong conformity with predictive models that account for curvature when the muscle is relaxed (Muramatsu et al. 2002) and are dependent upon fascicle angle and muscle thickness (an increase in fascicle angle will decrease fascicle length where no change in thickness occurs). Therefore, whilst automated analysis methods are time-efficient, complications may arise when examining fascicle length and, where available, future studies should implement extended field-of-view ultrasonography or sonograph multiple sites of the muscle to more accurately examine fascicle length (Franchi et al. 2017; Brusco et al. 2022). Additionally, only ISO appeared to improve handgrip strength, indicated by significantly lower values at pre-training but not post-training. This finding may indicate that the ISO-CONC and ISO-ECC did not fully comply with the handgrip protocol potentially because of dual-tasking (stair ascent/descent and handgrip) and thus, explain the comparable reductions in blood pressure. Whilst this is somewhat speculative, it is important to consider in future studies that implement simultaneous programmes as they may be time-efficient but potentially at the expense of adaptations. Consequently, stair ascent and descent do not appear to be sufficient stimuli to increase lower-limb strength of young adults, despite favourable adaptations in muscle structure; however, this may be enough stimulus to increase the strength of older adults, and thus, reduce falls risk and blood pressure concomitantly. Caution should be taken when prescribing simultaneous exercise modalities to older adults as the dual task nature may increase the immediate risk of falls, which may be particularly dangerous during stair climbing.

## Conclusion

The results indicate that all three training modalities lower SBP with no cumulative effects of stair walking in addition to handgrip training and that the simultaneous ISO-ECC group may have the potential to decrease DBP beyond that of the other training modalities despite lower objective and subjective demand. Moreover, stair walking was not sufficient stimulus to improve lower limb strength and resulted in marginal alterations in muscle structure, which only occurred in the concentric-biased group. Nonetheless, given the lower physiological capacity of some clinical populations, the stimulus from stair walking may be sufficient to improve muscular health as well as cardiovascular, however as clinical cohorts tend to face health-related barriers to exercise making them exercise-intolerant, stair descent may be favourable to prescribe. In light of these findings, further research in clinical populations who may be susceptible to greater changes, and in greater need of improvements in cardiovascular and muscular health, is needed.

## Data Availability

The datasets that support the findings of this study are openly available PURE at https://pure.northampton.ac.uk/en/datasets/dataset-simultaneous-isometric-hand-grip-and-stair-ascentdescent, or via the DOI at 10.24339/a9e1b0b0-8ab4-4eb8-b050-6da3b60b26e9.

## Abbreviations

ANCOVA: Analysis of covariance
ANOVA: Analysis of variance
BP: Blood pressure
CNAP: Continuous noninvasive arterial pressure
CR-10: Category-ratio
CTRL: Control
CV: Coefficient of variation
CVD: Cardiovascular disease
D: Dominant
DBP: Diastolic blood pressure
GM: Gastrocnemius medialis
GUI: Graphic user interface
HDL: High density lipoprotein
HR: Heart rate
ICC: Intraclass correlation coefficient
IHG: Isometric hand grip
IRT: Isometric resistance training
ISO: Isometric
Int$: International dollars
ISO-CONC: Isometric-concentric
ISO-ECC: Isometric-eccentric
LDL: Low density lipoprotein
MAP: Mean arterial pressure
MVC: Maximum voluntary contraction
ND: Non-dominant
PARQ +: Physical activity readiness questionnaire +
Q: Cardiac output
RBE: Repeated bout effect
RPE: Rate of perceived exertion
RTD: Rate of torque development
SBP: Systolic Blood Pressure
SD: Standard deviation
SE: Standard error
SV: Stroke volume
SVR: Systemic vascular resistance
USA: United States of America
VL: Vastus lateralis
Wk: Week
Int$: International dollars
